# ﻿Pollen and morphometric analysis reveal *Solanumtavinuuyuku* (Solanaceae), a new dioecious species from Mesoamerican Solanumsect.Anarrhichomenum

**DOI:** 10.3897/phytokeys.255.140014

**Published:** 2025-04-04

**Authors:** Jacob Bryant, Mariana Vazquez-Alonso, Eric J. Tepe

**Affiliations:** 1 Department of Biological Sciences, University of Cincinnati, Cincinnati, Ohio 45221, USA University of Cincinnati Cincinnati United States of America; 2 Department of Botany, Connecticut College, New London, Connecticut 06320, USA Connecticut College New London, Connecticut United States of America

**Keywords:** Dioecy, morphometrics, Mexico, palynology, Potato clade

## Abstract

*Solanumtavinuuyuku*, of the Sierra Madre del Sur ecoregion of South-Central Mexico, is a viny, node-rooting species of the Potato clade, in the subclade Solanumsect.Anarrhichomenum. *Solanumtavinuuyuku* is distinguished from its relatives by possessing long and narrow, lanceolate, ovate to falcate, 1–3-foliate leaves with (5–)7–10 secondary veins; large, persistent pseudo-stipules found at nearly every node on herbarium material; and white, violet-tinged corollas particularly pronounced in the floral bud. *Solanumtavinuuyuku* is functionally dioecious, producing functional, tricolporate pollen and non-functional, inaperturate pollen in short and long-styled flowers, respectively, joining its close relative *S.appendiculatum* as one of only two documented dioecious species in the entire Potato clade. Separation of *S.tavinuuyuku* from its closest ally, the simple-leaved *S.ionidium*, is based on morphometric and geographic evidence presented here.

## Abstract (fr)

*Solanumtavinuuyuku*, de la ecorregión de la Sierra Madre del Sur en el centro-sur de México, es una especie trepadora con raíces adventicias en los nodos que pertenece al clado de la papa, en el subclado *Solanum* sección *Anarrhichomenum*. *Solanumtavinuuyuku* se distingue de sus parientes por poseer hojas largas y estrechas, lanceoladas, ovadas a falcadas, de 1 a 3 foliolos con (5–)7 a 10 venas secundarias; grandes pseudoestípulas persistentes encontradas en casi todos los nodos en material de herbario; y corolas violetas, particularmente pronunciadas en el capullo floral. *Solanumtavinuuyuku* es funcionalmente dioica, produciendo polen tricolporado funcional y polen inaperturado no funcional en flores de estilo corto y largo, respectivamente, uniéndose a su pariente cercano *S.appendiculatum* como una de las únicas dos especies dioicas documentadas en todo el clado de la papa. La separación de *S.tavinuuyuku* de su aliado más cercano, *S.ionidium* de hojas simples, de las cuales muchas muestras fueron identificadas anteriormente, se basa en evidencia morfométrica y geográfica presentada aquí.

## ﻿Introduction

*Solanum* L. is one of the most species-rich and diverse genera of the angiosperms, encompassing ca.1200 species, and is the largest genus in the Solanaceae (Frodin 2004; Gagnon et al. 2022; Hilgenhof et al. 2023; Messeder et al. 2024; Moonlight et al. 2024). Apart from eminent crop species, potato, tomato and eggplant, the genus also includes many under-studied groups and new species continue to be discovered (e.g. Särkinen et al. (2015); Martine et al. (2016a, 2016b); Särkinen and Knapp (2016); Lacey and Martine (2017); Gouvêa et al. (2018, 2019, 2020); McDonnell et al. (2019); McClelland et al. (2020); Knapp et al. (2022); Tovar and Giacomin (2022); Williams et al. (2022); Tovar et al. (2024)).

Within *Solanum*, 12–13 major lineages have been resolved using modern molecular approaches (Weese and Bohs 2007; Särkinen et al. 2013; Gagnon et al. 2022). A total revision of the entire genus has not been undertaken since Candolle’s (1852)*Prodromus*, but progress is being made to that end by many authors, including by the team behind the Solanaceae Source web resource (www.solanaceaesource.org). Through that initiative, systematic work on the genus has advanced considerably, with workers revising infrageneric groups across the *Solanum* phylogeny (e.g. Levin et al. (2006); Tepe and Bohs (2011); Stern et al. (2013); Wahlert et al. (2014); Clark et al. (2015); Aubriot et al. (2016); Knapp et al. (2019); Spooner et al. (2016); Vorontsova and Knapp (2016)). One of the largest lineages of the ‘non-spiny’ species of *Solanum* is the Potato clade, comprising 12–13 well-supported subclades including the lesser-known and monophyletic Solanumsect.Anarrhichomenum (Tepe et al. 2016). Solanumsect.Anarrhichomenum has not been the subject of a comprehensive taxonomic revision and many previous taxonomic works have not recognized it as a single cohesive group. In his monograph of the wild potatoes, Correll (1962) organized some species of Solanumsect.Anarrhichomenum Bitter under *Solanum* series *Appendiculata* Rydb. (of sect. Basarthrum Bitter), but excluded the Mexican species *S.ionidium* Bitter. Nee (1993) was the first to suggest kinship between *S.appendiculatum* Dunal and *S.ionidium* and placed *S.ionidium* with the other four traditionally recognized species in Mesoamerica of what is now recognized as Solanumsect.Anarrhichomenum (*S.appendiculatum*, *S.skutchii* Correll, *S.tacanense* Lundell, *S.subvelutinum* Rydb. and *S.ionidium*). These Mesoamerican species make up a species complex defined by their morphological similarity to, and common ancestry with, the more well-known *S.appendiculatum* (Correll 1962; Nee 1999; Murillo-Pérez and Rodríguez 2021) and the new species described in this paper joins this group. More recent studies have clarified sectional limits and workers have contributed additional South American species, *S.complectens* M.Nee and G.J.Anderson (Nee et al. 2006) and *S.baretiae* Tepe (Tepe et al. 2012) to the clade. Species of this section are defined by their scandent, viny habit, rooting at the nodes, orange–red globose fruits and a single or, more rarely, paired, but strongly anisophyllous pseudo-stipules at each node along the stem (Tepe et al. 2016).

*Solanumappendiculatum*, a close relative of the new species proposed here, was the first dioecious species to be discovered within *Solanum* and has been the focal interest of many studies investigating the evolution of dioecy in *Solanum* (Anderson and Gensel 1979; Anderson and Levine 1982; Zavada and Anderson 1997; Wu et al. 2021). Staminate and pistillate plants of *S.appendiculatum* were originally described as three distinct taxa separated by differences in floral morphology (i.e. filament connation and style length) (Correll 1962), but were later determined to be a single dioecious species, based on cross compatibility and biosystematic studies in a series of landmark papers (e.g. Anderson and Gensel (1979); Anderson and Levine (1982)). Taken from the conclusions of these investigations are a series of morphological indicators that can be reliably used to determine the presence of dioecy in *Solanum*, even when cryptic, as in *S.appendiculatum*. Common to most known dioecious *Solanum* is stylar dimorphism and the production of inaperturate, non-functional pollen in the anthers of long-styled flowers and functional, tricolporate pollen in short-styled flowers (Anderson and Levine 1982; Anderson and Symon 1989; Anderson et al. 2015).

The Mesoamerican Solanumsect.Anarrhichomenum species complex remains poorly understood and is currently under revision by the authors. This study reports some of the results gleaned from ongoing systematic work on the complex and presents *S.tavinuuyuku*, a morphologically unique, dioecious *Solanum* species from Mexico, a center of diversification for Solanaceae (Rodríguez 2004; Villaseñor 2016) and the first new species of Mesoamerican Solanumsect.Anarrhichomenum to be described in over 70 years. Alongside the morphological and geographical analyses, we report the results from these investigations into the reproductive biology of *S.tavinuuyuku*, following the invaluable work of Anderson and colleagues (Anderson and Levine 1982; Anderson and Symon 1989; Zavada and Anderson 1997; Anderson et al. 2015).

## ﻿Materials and methods

### ﻿Sampling and measurement

Observations were based on examination of 38 herbarium specimens on loan and/or specimen images from European and Central, South and North American Herbaria (AAU, ARG, B, BH, BM, BR, C, CORD, F, G, GH, LL, MEXU, MICH, MO, NY, OAX, SERO, UC, UGAC, US, WIS, XAL). Physical specimens were measured with a standard ruler and, for smaller structures, a 1-cm micro-ruler with 0.1 mm marks (Ted Pella, Inc., Redding, CA) under a Leica M80 dissecting microscope. Three specimens of the new species were observed and measured using digitized collections from BM, MEXU, NY (Suppl. material 1). In total, all 20 specimens of the eight known collections of *S.tavinuuyuku* were sampled for this study, along with 18 collections of *S.ionidium*, and 12 collections of *S.appendiculatum* (Suppl. material 1). Specimens of *S.appendiculatum* were limited to collections that were sympatric with *S.tavinuuyuku* or were highly similar in leaf characteristics.

### ﻿Morphometrics

Factor Analysis of Mixed Data (FAMD) was conducted and visualized in Rstudio (R Core Team 2021) using the packages “FactoMineR” (Le et al. 2008) and “factoextra” (Kassambara and Mundt 2020), respectively, on a total of 15 morphological and two geographic variables (Suppl. material 2). FAMD was selected as the most appropriate tool for exploratory multivariate analysis over the more common PCA because it can incorporate mixed data regimes (Pages 2004). Of the total 15 variables, 12 morphological and one geographic variable were continuous measures and one morphological (corolla color) and one geographic variable (mountain range where specimens were collected) were categorical. Characters such as stamen length and width and style length were excluded from analysis due to their relationship to dioecy (Correll 1962; Anderson and Levine 1982; Anderson et al. 2015). On unifoliate specimens, the absence of lateral leaflets was represented by zeros in the data matrix. Highly co-linear variables such as leaf, leaflet and pseudo-stipule length and width are represented in our study by a ratio.

To prepare for FAMD, the data were first imputed and dimensions were visualized in a scree plot. The ten most influential variables contributing to each dimension were visualized as barplots. FAMD results were plotted in an ordination plot with 95% confidence interval (CI) ellipses based on a multivariate t-distribution grouped by taxonomic classification and a biplot of quantitative variables. Afterwards, multiple-comparison Dunn’s tests were conducted on all variables included in morphometric analysis using the base “FSA” package in R (Ogle et al. 2022) and visualized as a series of boxplots.

### ﻿Mapping

**Figure 1.**
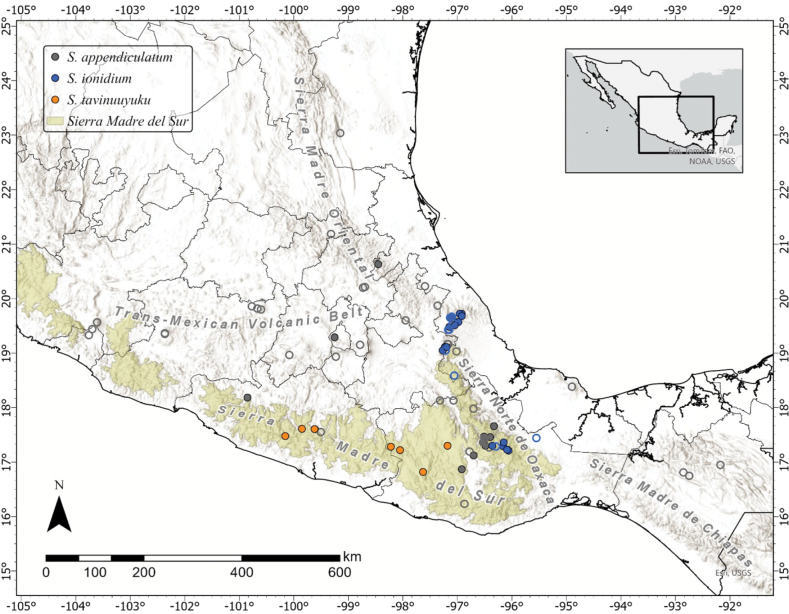
Distribution map of the three species of Solanumsect.Anarrhichomenum included in this study. The map is composed of collection locations of 82 combined accessions of *Solanumtavinuuyuku* (orange); *Solanumionidium* (blue) and *Solanumappendiculatum* (grey). Specimens included in the morphometric analyses are indicated by filled circles. Supplementary accessions are indicated by open circles. The Sierra Madre del Sur biogeographic region was mapped using shapefiles of biogeographic provinces provided in Morrone et al. (2017).

All accessions used in the morphological study were then mapped alongside an additional 46 accessions of *S.appendiculatum* and *S.ionidium* to visualize the distribution of *S.tavinuuyuku* in comparison to those of its relatives *S.appendiculatum* and *S.ionidium* in Mexico (Fig. 1). Thirty-seven of these collections were of *S.appendiculatum*, seven were derived from digital images provided by Mexican herbaria (OAX and UGAC) and the rest were directly examined by the authors (Suppl. material 1). Nine additional accessions of *S.ionidium* were also included, all of which were received as digital images from Mexican herbaria (XAL and OAX) (Suppl. material 1). In total, combining supplementary accessions and those included in morphometric analyses, 82 data points were mapped, encompassing all known collections of *S.tavinuuyuku*, along with selected accessions of *S.appendiculatum* and *S.ionidium*. Maps were constructed in ArcGIS Pro using reference coordinates from herbarium labels or georeferences derived from location descriptions using the online GEOLocate application (Rios and Bart 2010).

### ﻿Palynology

Pollen grains were retrieved from all available herbarium collections (Suppl. material 1) to investigate the presence of dimorphic pollen in *S.tavinuuyuku*. Three specimens represented staminate, short-styled plants, while the remaining two were long-styled, pistillate plants. Pollen grains were extracted with permission by removing individual anthers from flowers in debris packets on herbarium specimens and then perturbing the anther to release the grains, scraped from a presenting anther or retrieved by inserting an insect pin into the distal end of the anther. Pollen grains were then either mounted directly in a 1:1 glycerine-water solution or treated using a modified acetolysis procedure (Erdtman 1960; Bryant 2025) Both untreated and treated pollen samples were viewed using phase contrast and scanning electron microscopy (SEM) with a Nikon Labophot-2 phase contrast scope and Apreo 2 SEM, respectively.

Throughout this paper, specimen barcodes and accession numbers are cited in brackets. Barcode numbers are listed after an herbarium acronym, whereas accession numbers include the number only. When digital collections of the new species were used, these collections are indicated by the ‘[photo]’ notation.

## ﻿Results

### ﻿Morphometrics

**Figure 2.**
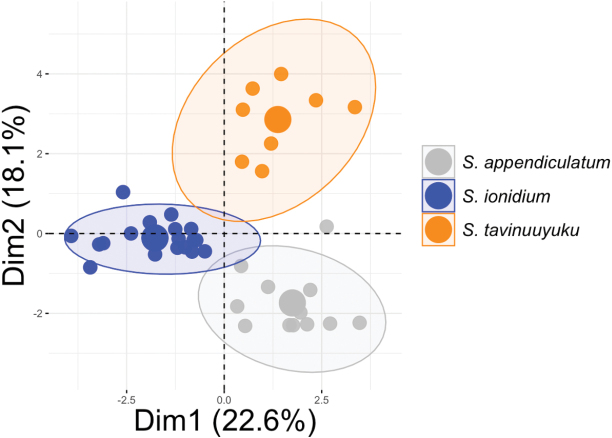
Factor analysis of mixed data (FAMD) ordination plot of *Solanumtavinuuyuku* and sympatric or near sympatric species from Solanumsect.Anarrhichomenum with 95% CI ellipses.

**Figure 3.**
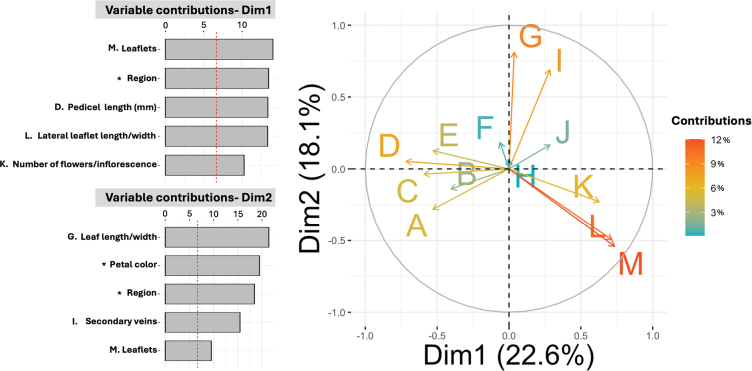
Barplots and a biplot of the contributions of influential variables in FAMD. Barplots represent the variable contributions of the top five most influential categorical and quantitative variables in FAMD analysis. Influential quantitative variables are labelled **A –M** and can be found in the biplot, while variables marked with an asterisk (*) represent influential categorical variables, which are not represented in the biplot. Peduncle length (cm) (**A**); pseudo-stipule length/width ratio (**B**); calyx lobe width (mm) (**C**); pedicel length (cm) (**D**); flower diameter (**E**); calyx length (mm) (**F**); leaf length/width ratio (**G**); petiole length (cm) (**H**); number of secondary veins (**I**); elevation (m) (**J**); flowers per inflorescence (**K**); lateral leaflet length/width ratio (**L**); number of leaflets (**M**).

**Figure 4.**
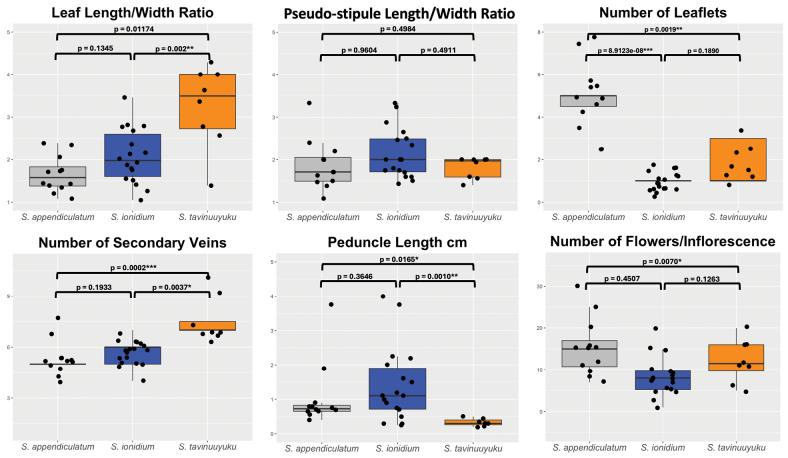
Boxplots of important morphological characters distinguishing *Solanumtavinuuyuku* from *Solanumappendiculatum* and *Solanumionidium.* The upper and lower bounds of the box represent the upper and lower quartiles, respectively. The middle line within the box represents the median value. Maximum and minimum values are represented by the extent of the upper and lower “whiskers”. Bars above each box within each graph constitute pairwise comparisons between species. P-values are derived from the results of multiple comparison Dunn’s tests and were adjusted using Bonferroni correction (Suppl. material 2). Asterisks represent a significant result in Dunn’s tests. A single asterisk (*) = p < 0.05; double asterisks (**) = p < 0.005; triple asterisks (***) = p < 0.0005.

For multivariate species delimitation, the first two dimensions were retained in FAMD analysis accounting for 40.7% of total variance in the data. Results revealed a distinct separation of *S.tavinuuyuku* from its close relatives *S.ionidium* and *S.appendiculatum* in morphospace (Fig. 2). Variables contributing to the separation included number of leaflets, region of collection, pedicel length, lateral leaflet length/width ratio, number of flowers per inflorescence, leaf length/width ratio, corolla color and number of secondary veins (Fig. 3). All accessions of *S.tavinuuyuku* clustered together around positive eigenvalues along Dim 1 and Dim 2 and most collections fell within or near the 95% CI ellipse for that species (Fig. 2). Conversely, *S.ionidium* collections clustered around negative eigenvalues along Dim 1 and demonstrated a near complete separation from *S.tavinuuyuku* (Fig. 2). *Solanumappendiculatum* occupied an intermediate space between *S.tavinuuyuku* and *S.ionidium* along Dim 1, but fell within negative eigenvalues along Dim 2 (Fig. 2), differing from both its relatives in leaflet dimensions, number of leaflets, leaf length/width ratio and flowers per inflorescence (Fig. 3). Non-parametric Dunn’s tests for multiple comparisons supported differences between the new species and one or both relatives in seven of the fifteen variables included in FAMD (Fig. 4; Suppl. material 2). Of these, *S.tavinuuyuku* differed (p < 0.05) from *S.ionidium* in four morphological variables and from *S.appendiculatum* in six morphological variables, as well as the region of collection between both species (Suppl. material 2).

### ﻿Palynology

**Figure 5.**
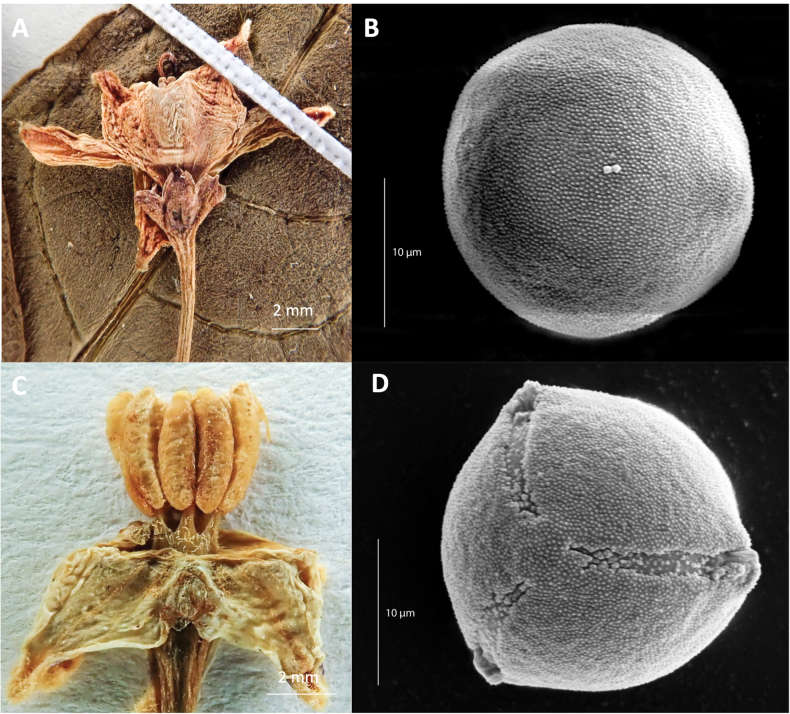
Dimorphic flowers and pollen grains representative of the pistillate and staminate sex of *Solanumtavinuuyuku*. **A** Pistillate flower (Croat 45261a) **B** SEM of inaperturate pollen from a functionally pistillate flower (Calzada 19434) **C** staminate flower (Rzedowski 159) **D** SEM of tricolporate, aperturate pollen from a functionally staminate flower (Calzada 19831) at 3,500× magnification.

Inaperturate pollen of rounded shape (20–[23.3]–25 µm × 21.00–[23.17]–27.00 µm), was observed in two of the five samples included in this palynological study (*Croat 45517* and *Calzada 19434*) and convexly triangular, tricolporate pollen (20–[23.33]–25 µm × 21.00– [23.17]–27.00 µm) in the remaining three (*Calzada 19831*, *Rzedowski 159* and *Tenorio 1414*). The inaperturate condition was only observed in the anthers of long-styled flowers, whereas tricolporate pollen was found in the anthers of short-styled plants (Fig. 5). The combination of stylar and pollen dimorphism (inaperturate and aperturate) suggests that *S.tavinuuyuku* is likely to be dioecious.

## ﻿Taxonomic treatment

### ﻿ Solanum tavinuuyuku

Taxon classificationPlantaeSolanalesSolanaceae

J.M.Bryant & Tepe sp. nov.

0037EDE4-18D4-5449-A17A-3AE469B0549C

urn:lsid:ipni.org:names:77359685-1

Fig. 6

#### Diagnosis.

*Solanumtavinuuyuku* resembles *Solanumionidium* Bitter, but is distinguished from that species by its long and narrow, lanceolate, ovate to falcate, simple to 3-foliate leaves with 7–10 secondary veins, large and persistent pseudo-stipules, sparse to dense pubescence and white to violet-tinged corolla color that is especially pronounced in the floral bud.

#### Type.

Mexico. Oaxaca • Distr. Santiago Juxtlahuaca. Mpio. Santiago Juxtlahuaca. Loc. Puerta de Luz, hacia la torre microondas, entrada por Santa Rosa, via San Miguel Cuevas a El Manzanal, 17.13°N, 98.03°W, 2405 m elev., 18 Apr 1995 (fl), *J. I. Calzada 19831* (holotype: MEXU! [892615]; isotypes: LL! [00218235], NY! [NY00751674]).

#### Description.

Vine, trailing along ground or climbing on other vegetation to 10 m or more, rooting at the nodes. ***Stems*** woody, sparsely to densely pubescent with long, transparent to pale tawny, unbranched, eglandular, multicellular simple hairs (termed finger-hairs in Seithe and Anderson (1982)), 0.5–0.8 mm long, more densely pubescent at the nodes, the stems often with white lenticels. ***Sympodial units*** plurifoliate, not geminate. ***Leaves*** distichous, simple to 3-foliate pinnately compound, tending towards 3-foliate on juvenile vegetative shoots and simple on mature shoots, the blades 2–14 × 1–5 cm, chartaceous, the margins flat to somewhat revolute, not ciliate, lamina sparsely to densely pubescent to glabrescent abaxially and adaxially, but on average, more densely pubescent adaxially, on veins and on developing leaves, the rachis and petiolules densely pubescent with multicellular finger hairs; simple leaves 3.8–11 cm × 1.5–3.5 cm, ovate, lanceolate to narrowly elliptic in shape, somewhat falcate on some specimens, the bases acute to rounded, symmetrical to asymmetrical, the apices acute to acuminate, the acumen 0.3–1 cm long, venation pinnate, (5–)7–10 pairs of secondary veins, secondary veins curved; compound leaves include an apical leaflet and a single pair of lateral leaflets, the distal lateral leaflet often smaller than its counterpart or completely absent; apical leaflets of compound leaves 5.5–9 cm × 2–3 cm, substantially larger than lateral leaflets, ovate to lanceolate, the bases acute, rounded to nearly truncate, symmetrical to asymmetrical, the apices acuminate, the petiolules 1.5–2.5 mm; lateral leaflets 2–4.5 cm × 0.5–2 cm, elliptic to ovate, the apices acute, the petiolules nearly lacking to 1 mm; interjected leaflets absent; petioles 0.3–5(–8.6) cm, densely pubescent. ***Pseudo-stipules*** present at nearly every node, single, persistent, 3–5 × 6.5–10 mm, reniform or more rarely subovate, the apices sharply acute, pubescence resembling that of the leaves. ***Inflorescences*** terminal corymbose-cymes, 2–3 × branched, 1.5–2.2 cm × 2–2.5 cm in flower, 3.5–6 cm × 2.5–4 cm in fruit, with 6–15 flowers distanced 0.5–3 mm apart, the axes glabrous, glabrescent or less frequently moderately pubescent, the peduncle 1–5(–11) mm, becoming extra-axillary with added sympodia, occasionally presenting a single bract-like leaf opposite the branching point; pedicels 0.2–1.2 cm in flower, 1–1.5 cm in fruit, articulated at the base, 0.5 mm wide across the base, expanded distally in flower and fruit up to 2 mm, moderately pubescent in flower with scattered, long, slender, finger hairs, 0.3–0.8 mm, glabrescent to glabrous in fruit. ***Flowers*** pentamerous, apparently perfect, but dimorphic, morphologically complete, with anthers from pistillate flowers producing inaperturate pollen. ***Calyx*** campanulate, 1.5–2.5 mm × 1.5–3(–6) mm in developing buds, 3–3.5 mm × 3–4 mm on mature flowers, the tube 1–1.5 mm long in flower and fruit, 5-lobed, the lobes triangular with acute to apiculate apices, 1–1.5 mm × 1–1.5 mm in flower, 1.5–2.5 mm × 1.5–2.5 mm in fruit, the margins somewhat scarious, the lobes with short, stocky, clustered pubescence on the apices, 0.02–0.3 mm and often sparsely to densely pubescent with long, slender, appressed, finger hairs, 0.3–0.7 mm, across the entire calyx, glabrescent in fruit. ***Corolla*** 0.8–1.2 cm in diameter, stellate, 5-lobed, reflexed or flat at anthesis, the tube 1.5–2.5 mm, the lobes 3.5–6 mm × 2–2.5 mm, ovate to triangular, acute at apices, puberulent on apices and along the distal half to third of the margins, violet with white along petal margins (as visible on herbarium material), violet color pronounced on developing buds. ***Stamens*** equal, with filaments 0.3–1 mm long, free to partially fused to half the entire filament length, pubescent; anthers 2.8–3.1 mm × 1–1.2 mm, oblong with retuse apices, cordate at the bases, connivent, yellow, with large apical pores, developing into introrsely dehiscent longitudinal slits with age. ***Ovary*** glabrous; the style on short-styled, staminate flowers 1–3 mm or nearly absent, far shorter than stamens, cylindrical, the stigma slightly clubbed or truncate; the style on long-styled, pistillate flowers 5.5–6 mm, exserted above the stamens, curved at the tip, cylindrical, the stigma capitate. ***Fruits*** 0.3–1 cm in diameter, globose, green when immature to bright orange-red at maturity, glabrous. ***Seeds*** 3.5–4 mm × 2.2–3 mm, flattened, rounded to teardrop-shaped, with a 0.2–0.5 mm wide wing around the margins, the thickened part of the seed 2.5–3 mm × 2–2.5 mm, rounded to oval-shaped, lenticular, light to medium brown, the surface smooth, the wing yellowish-tan to translucent near the margins, with radial striations, 15–20 seeds per fruit.

**Figure 6.**
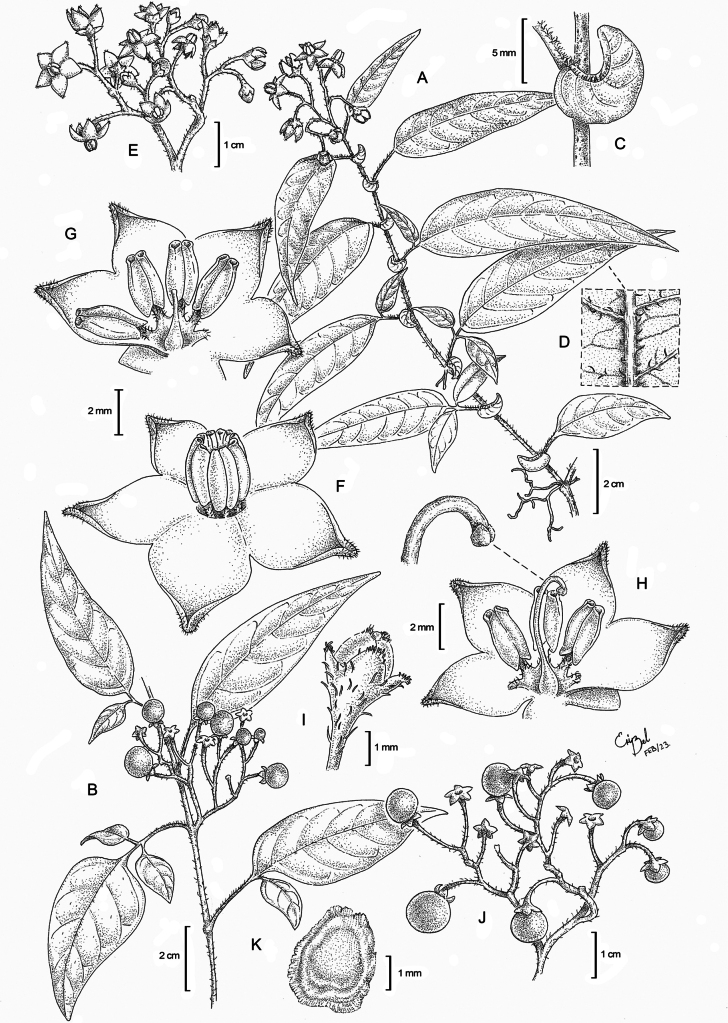
*Solanumtavinuuyuku***A** habit of flowering branch **B** habit of fruiting branch **C** pseudo-stipules with pubescence **D** abaxial leaf surface showing pubescence on the mid- and secondary veins **E** inflorescence **F** staminate flower **G** staminate flower with staminal column opened to reveal pistil **H** pistillate flower and detail of the pistil, with stigma magnified **I** calyx with pubescence **J** infructescence with mature berries **K** seed. [**A, C–G** drawn from Calzada 19831 **B, J** drawn from Calzada 19434 **H** drawn from Croat 45517 **I** drawn from Croat 45261a **K** drawn from Rzedowski 159]. Art by Ericka Belén Cortez Castro.

#### Distribution and ecology.

**Figure 7.**
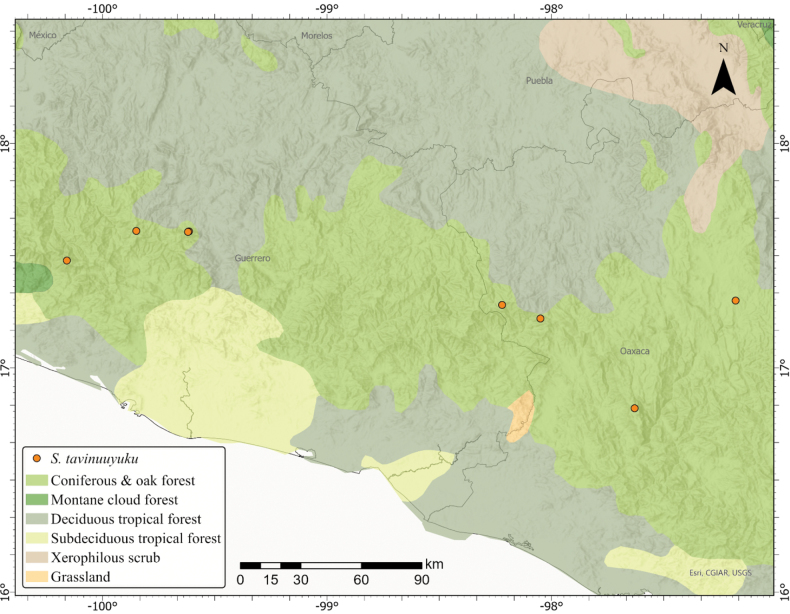
Distribution of *Solanumtavinuuyuku* in Mexico in the States of Oaxaca and Guerrero, within Coniferous and oak forest potential vegetation (Rzedowski 1990).

*Solanumtavinuuyuku* appears to be endemic to south-central Mexico and has been collected in extreme western Oaxaca (Mixteca and Sierra Sur Regions) through central Guerrero (La Montaña and Centro Regions). These areas are a part of the Sierra Madre del Sur ecoregion, where *S.tavinuuyuku* grows in the understory of pine-oak forests and montane cloud forests, from 1700 to 2840 m in elevation (Fig. 7).

#### Phenology.

*Solanumtavinuuyuku* appears to flower year-round. Flowering specimens have been collected January, April, August, October and December; fruiting specimens are from January, August and October.

#### Etymology.

The epithet *tavinuuyuku* is of Mixtec origin and is derived from a combination of the Mixtec words *tavi nuu yuku*, which can be roughly translated to mean “found growing in the mountains”. We acknowledge that the very act of naming of natural objects is political. Taxonomists have and are still often guilty of acquiring these natural entities as their own intellectual possession, dictating, in a sense, the language in which we communicate about the world we inhabit. Nonetheless, it is our intent that by choosing this name, we honor the contributions of all indigenous nations and peoples in documenting and conserving local biodiversity, particularly the Mixtec people who inhabit these landscapes. Although we were not able to discover a local name for this plant, we propose an epithet sourced from a language familiar to the people who have undoubtedly interacted with it for millennia.

#### Conservation statement.

IUCN Red List Criteria (IUCN 2022) suggest a status of vulnerable (VU) for *S.tavinuuyuku*, based on criteria B1 a, b (i, iii) with an extent of occurrence (EOO) of ca. 18,582 km^2^. The species is known from only eight localities and is either quite rare or particularly under-collected (Fig. 1). The Sierra Madre del Sur ecoregion is composed primarily of rugged terrain, can be quite remote and thankfully is a conservation priority in Mexico (Arriaga Cabrera et al. 2000). However, the forest habitats where *S.tavinuuyuku* grows are under threat from ranching and agricultural activities in the area (Toledo-Aceves et al. 2011) which may impact the demographic and ecological stability of the species in the future if current trends continue.

#### Specimens examined.

**Mexico. Guerrero** • 2 km al NE del Campamento El Gallo; estribaciones SW del Cerro Teotepec, 2650 m elev., 27 Jan 1965 (fl, fr), *J. Rzedowski and McVaugh 159* (CORD, F, MEXU [photo]) • Mun. Eduardo Neri, along highway between Milpillas (on Highway 95) and Atoyac de Alvarez, 3.7mi W of turn-off on to road to Chichihualco, 17.08°N, 99.73°W, 2325 m elev., 14 Jan 1979 (fl, fr) *T.B. Croat 45621-a* (BM [photo], F, MO, NY, US) • Along road between Milpillas at Highway 95 and Atoyac de Alvarez (near Highway 200) 3.5 miles west of junction with road to Chichihualco, 2425 m elev., 12 Jan 1979 (fl), *T.B. Croat 45517* (MO) • 15 km al E de Pto. del Gallo, Carr. Filo de Caballo. Mpio. Chichihualco, 3090 m elev., 18 Aug 1982 (fl), *L. Hernandez S. and C. Romero de T. 1414* (MEXU [photo], NY). **Oaxaca** • Distr. Santiago Juxtlahuaca, Mpio: San Martin Peras, 17.18°N, 98.11°W, 2565 m elev., 10 Oct 1994 (fl, fr), *J.I. Calzada 19434* (LL, MEXU [photo], NY) • Mun. San Martin Peras, Loc. Dt. de Juxtlahuaca. A 3 km de la desviación a San Martin Peras, hacia Coicoyán de las Flores, 17.28°N, 98.22°W, 3 Dec 1992 (fl) *Torres et al. 14098* (MEXU [photo]) • Distr. Putla, Mpio. Santa Cruz Itundujia, A 7.37 km en LR (SE) de Santa Cruz Itundujia, 16.82°N, 97.63°W, 2865 m elev., 1 Aug 2008 (fl, fr) *Gutierrez et al. 3075* (MEXU [photo], SERO [photo]).

### ﻿Key to species of Solanumsect.Anarrhichomenum in Mexico

**Table:** 

1	Leaves unifoliate	**2**
–	Leaves (2–)3–7 foliate	**3**
2	Leaves broadly ovate with bases cordate to rounded, symmetrical and secondary veins 5– 7. Corollas white. Collected in the Sierra Norte de Oaxaca in the Sierra Madre del Sur ecoregion and in the Sierra Madre Oriental on Mexico’s gulf coast	***S.ionidium***
–	Leaves lanceolate to narrowly elliptic with bases rounded, asymmetrical to symmetrical and secondary veins (5–)7–10. Corollas white, often tinged with violet. Collected in the southern mountains of the Sierra Madre del Sur ecoregion	***S.tavinuuyuku***
3	Anthers oblong with retuse apices	**4**
–	Anthers oblong with aristate apices	***S.skutchii***
4	Apical leaflet with secondary veins (5–)7–10, over double the length of the lateral leaflets	***S.tavinuuyuku***
–	Apical leaflet with secondary veins 5–7, slightly larger than lateral leaflets or roughly equivalent in size and shape	***S.appendiculatum* (incl. *S.subvelutinum* , *S.tacanense*)**

## ﻿Discussion

*Solanumtavinuuyuku* is the first species to be added to Mesoamerican Solanumsect.Anarrhichomenum in nearly 70 years and joins the *S.appendiculatum* species complex, a morphologically and taxonomically complex group of understudied vines. *Solanumtavinuuyuku* is restricted to the unique Sierra Madre del Sur ecoregion in south-central Mexico (Aragón-Parada et al. 2021) that spans the political State of Guerrero and south-western Oaxaca and is the only 1–3 foliate species of Solanumsect.Anarrhichomenum in that area. *Solanumtavinuuyuku* is also of considerable interest as it is only the second documented dioecious species in the Potato clade and amongst the greater so-called ‘non-spiny’ *Solanum* (Weese and Bohs 2007), apart from *S.appendiculatum*.

### ﻿Comparative morphology and geography of sympatric species

Most collections of *S.tavinuuyuku* were originally identified as *S.ionidium*, which was once believed to be the only unifoliate species amongst Mesoamerican Solanumsect.Anarrhichomenum. Superficial similarities in the leaves and the lack of sufficient systematic study of this group have made it easy to misidentify unifoliate collections of *S.tavinuuyuku*, especially when working with herbarium material. *Solanumionidium* has not been the subject of any focused taxonomic studies since Bitter initially described the species (Bitter 1913) and its placement in Solanumsect.Anarrhichomenum is relatively recent (Nee 1999). Despite the similarities in leaf morphology, however, these two species are quite distinct.

Morphologically, *S.tavinuuyuku* differs from *S.ionidium* in five out of the fifteen morphological characters assessed in this study. Long and narrow leaves, (5–)7–10 secondary veins, shorter peduncles (ca. 0.3 cm in *S.tavinuuyuku* vs. ca. 1.5 cm in *S.ionidium*) and violet-tinged flowers can reliably be used to distinguish the new species from *S.ionidium* and these characters favoured separation in morphometric analyses. *Solanumionidium* is also differentiated from the new species by possessing exclusively unifoliate leaves, ciliate leaf margins, vegetative parts with glabrous or sparsely pubescent indument and longer pedicels on average (ca. 1.3 cm in *S.ionidium* vs. 0.9 cm in *S.tavinuuyuku*).

Furthermore, *S.tavinuuyuku* is not sympatric with *S.ionidium*, which inhabits only the north-eastern extent of the Sierra Madre del Sur in northern Oaxaca (Sierra Norte de Oaxaca), but has principally been collected throughout the Sierra Madre Oriental mountains in the State of Veracruz (Fig. 1). *Solanumtavinuuyuku* occupies pine-oak habitats in the southern mountains of the Sierra Madre del Sur of south-western Oaxaca and grows throughout the mountains that span the State of Guerrero (Fig. 7), but does not cross the Valley of Oaxaca into Sierra Norte de Oaxaca (Fig. 1).

*Solanumtavinuuyuku* does grow in sympatry with its close relative, *S.appendiculatum* (Fig. 1), and may be confused with that species on specimens with primarily 3–foliate leaves or that lack lateral leaflets (which happens very rarely). However, it is very rare to find a collection of *S.appendiculatum* that has apical leaflets as long as a unifoliate leaf or apical leaflet of *S.tavinuuyuku*. Moreover, the apical leaflets of *S.appendiculatum* have longer petiolules, 5–6 secondary veins on average (vs. 7–10 in *S.tavinuuyuku*) and are almost always elliptic–ovate with symmetrical bases (vs. ovate–lanceolate leaves with asymmetrical–symmetrical bases as in *S.tavinuuyuku*).

On 3-foliate specimens, *S.tavinuuyuku* is distinguished by very long apical leaflets and smaller lateral leaflets (ca. 0.51 cm × ca. 0.25 cm in *S.tavinuuyuku* vs. ca. 1.94 cm × ca. 0.82 cm in *S.appendiculatum*) that are less than half the length of the apical leaflet on average. Some compound leaves may only have a single lateral leaflet paired with a small indentation or gap on the apical leaflet giving the leaf an asymmetrical appearance, a feature which is not apparent in *S.appendiculatum*. Due to their small size, the lateral leaflets can be overlooked on herbarium material. This is never the case with *S.appendiculatum*, which has lateral leaflets roughly equivalent to, or only slightly smaller than the apical leaflet (the apical leaflet is ca. 2.5× longer than the lateral leaflet in *S.tavinuuyuku* vs. the apical leaflet ca. 1.20× longer than the lateral leaflet length in *S.appendiculatum*) and is clearly a compound-leaved species of 3–7 leaflets (vs. the often unifoliate presentation of *S.tavinuuyuku*). *Solanumappendiculatum* does often produce violet-tinged flowers in populations collected in the Central American Volcanic Arch, but produces mostly white flowers in the northern part of its range, including the Sierra Madre del Sur populations. In sympatric populations, *S.appendiculatum* can be differentiated from *S.tavinuuyuku* by corolla color, both in the floral bud and in flower, longer peduncles (ca. 0.33 cm in *S.tavinuuyuku* vs. ca. 1.05 cm in *S.appendiculatum*) and smaller pseudo-stipules (ca. 6.94 mm × ca. 3.83 mm in *S.tavinuuyuku* vs. ca. 5.32 mm ×. ca. 3.02 mm in *S.appendiculatum*).

*Solanumtavinuuyuku* also grows in sympatry with *S.morelliforme* Bitter & Muench and some collections of *S.tavinuuyuku* have been mistaken for that species, particularly due to its epiphytic, viny habit and long, lanceolate, simple leaves. However, *S.morelliforme* is a member of Solanumsect.Petota (Spooner et al. 2004), the tuber-bearing species of the Potato clade, which can easily be separated from Solanumsect.Anarrhichomenum by their tubers, articulation of the pedicel above the base and lack of adventitious roots at the nodes.

### ﻿Dioecy in *Solanum*

Dioecy has been identified in four of the thirteen infrageneric major and minor clades of *Solanum*, comprising only ca. 16 species to date (Anderson et al. 2015; Williams et al. 2022; Gagnon et al. 2022; Hilgenhof et al. 2023), with the majority occupying the so-called “spiny” Solanumgroup orsubg.Leptostemonum Bitter (Knapp et al. 1998; Martine et al. 2006, 2009, 2019; Anderson et al. 2015). All known dioecious species in *Solanum* are morphologically hermaphroditic, though some species are strongly dimorphic and others are cryptic, as is the case with *S.appendiculatum* (Anderson and Levine 1982). In a collection of ground-breaking studies, Anderson and collaborators provided a suite of morphological and reproductive indicators that are empirically strong, reliable lines of evidence that indicate dioecy in *Solanum* (Anderson and Gensel 1979; Anderson and Levine 1982; Levine and Anderson 1986; Anderson and Symon 1989; Mione and Anderson 1992; Zavada and Anderson 1997; Anderson et al. 2015). These studies, along with others (Knapp et al. 1998; Martine et al. 2009; Wu et al. 2021), have characterized functionally female plants as possessing flowers that are capable of bearing fruit with long styles exserted above the androecium, well-developed, capitate stigmas and anthers that produce non-functional inaperturate pollen or no pollen at all. Inaperturate pollen can be recognized by their rounded shape and lack of apertures (Anderson and Gensel 1979) and, in at least some species, are typically less nutritious and possess lower abundances of some amino acids essential for pollen tube development (Mattioli et al. 2012, 2018; Ndem-Galbert et al. 2021). The mechanical inhibition of pollen tube growth, resulting from the improper development of apertures and a reduced amino acid content, plays a key role in producing the feminizing phenotype of inaperturate pollen grains in dioecious *Solanum* species (Zavada and Anderson 1997; Ndem-Galbert et al. 2021). Functionally male plants, on the other hand, have flowers with short styles that are equal to or shorter than the androecium with underdeveloped, more cylindrical stigmas, that normally do not bear fruit with viable seeds. Pollen produced by these individuals is tricolporate and fully functional (Anderson and Levine 1982; Martine et al. 2016b) and has a convexly triangular shape in polar view (Anderson and Gensel 1979; Knapp et al. 1998).

The dioecious sexual system of *S.tavinuuyuku* was first suggested by the pattern of floral dimorphism described above. For example, two reliable collections with both flowers and fruits connected on the same branch have long-styled flowers and well-developed capitate stigmas (*Croat 45621-a* [BM, F, MO, NY, US], *Gutierrez 3075* [MEXU, SERO] and *Calzada 19434* [LL, MEXU, NY]). Pollen grains derived from the anthers of two similar representative pistillate plants (*Croat 45517* [MO] and *Calzada 19434* [LL, MEXU, NY]) completely lacked apertures (Fig. 6), resembling the rounded inaperturate grains from pistillate plants of *S.appendiculatum* (Anderson and Gensel 1979; Knapp et al. 1998). In contrast, short-styled flowering collections do not have fruits present on the same branch, have styles that do not exceed the androecium and have truncated and underdeveloped stigmas (*Calzada 19831* [LL, MEXU, NY], *Tenorio 1414* [MEXU, NY], and *Torres 14098* [MEXU]). Two of these three specimens (*Calzada 19831* [LL, MEXU, NY], and *Tenorio 1414* [MEXU, NY]) were available for direct observation (the other obtained as a digital image) and pollen sampled from these specimens all appeared tricolporate and convexly triangular (Fig. 6), much like the pollen of staminate plants of *S.appendiculatum* (Anderson and Gensel 1979; Knapp et al. 1998).

One sheet of *Rzedowski 159* (F) appears at first glance to violate the expected morphological pattern typical of dioecy in *S.lanum.* This exceptional specimen presents both a short-styled flowering shoot and a fruiting shoot. The shoots are mounted on the same herbarium sheet, but are disconnected. When examined, pollen grains sampled from both sheets of this collection rendered convexly triangular, tricolporate pollen, as would be expected from a short-styled, staminate flower. Taken together, it is highly unlikely that the flowering and fruiting shoot are from the same individual. It is not uncommon for collectors to intentionally take from a multitude of individuals representative of a population or unintentionally mount together two individuals that have become intertwined. Due to its anomalous presentation, inconsistency with the morphological pattern evident in the majority of *S.tavinuuyuku* specimens and pollen morphology, this collection is likely a mixture composed of a functionally male flowering and female fruiting individual, representing a population rather than an individual.

Although investigations into pollen viability and tube growth in inaperturate grains, seed set in short-styled flowers and genomic signatures of sex would be ideal for definitively determining dioecy (Anderson and Levine 1982; Mione and Anderson 1992; Anderson et al. 2015; Wu et al. 2021), the combination of floral and pollen dimorphism in a robust sample of known collections of this species is strong enough to confidently describe *S.tavinuuyuku* as dioecious.

## Supplementary Material

XML Treatment for Solanum tavinuuyuku

## ﻿Additional information

### Author ORCIDs

Jacob Bryant https://orcid.org/0009-0004-7462-8359

Mariana Vazquez-Alonso https://orcid.org/0000-0002-1285-5773

Eric J. Tepe https://orcid.org/0000-0002-8493-0736

### Data availability

All of the data that support the findings of this study are available in the main text or Supplementary Information.

## Supplementary materials

Supplementary material 1 Specimen accessions used in morphometrics, mapping, and dioecy studies Data type xlsx https://binary.pensoft.net/file/1299608This dataset is made available under the Open Database License (http://opendatacommons.org/licenses/odbl/1.0/). The Open Database License (ODbL) is a license agreement intended to allow users to freely share, modify, and use this Dataset while maintaining this same freedom for others, provided that the original source and author(s) are credited.Jacob Bryant, Mariana Vazquez-Alonso, Eric J. Tepe

Supplementary material 2 Post-hoc Dunn’s Tests results comparing morphological and geographic characters Data type xlsx https://binary.pensoft.net/file/1299609This dataset is made available under the Open Database License (http://opendatacommons.org/licenses/odbl/1.0/). The Open Database License (ODbL) is a license agreement intended to allow users to freely share, modify, and use this Dataset while maintaining this same freedom for others, provided that the original source and author(s) are credited.Jacob Bryant, Mariana Vazquez-Alonso, Eric J. Tepe

Supplementary material 3 Accession information in DarwinCore format Data type csv https://binary.pensoft.net/file/1299610This dataset is made available under the Open Database License (http://opendatacommons.org/licenses/odbl/1.0/). The Open Database License (ODbL) is a license agreement intended to allow users to freely share, modify, and use this Dataset while maintaining this same freedom for others, provided that the original source and author(s) are credited.Jacob Bryant, Mariana Vazquez-Alonso, Eric J. Tepe

Supplementary material 4 Character matrix of morphological variables used in multivariate statistical analyses Data type csv https://binary.pensoft.net/file/1299611This dataset is made available under the Open Database License (http://opendatacommons.org/licenses/odbl/1.0/). The Open Database License (ODbL) is a license agreement intended to allow users to freely share, modify, and use this Dataset while maintaining this same freedom for others, provided that the original source and author(s) are credited.Jacob Bryant, Mariana Vazquez-Alonso, Eric J. Tepe
